# Valuable Surgical Discovery

**Published:** 1859-10

**Authors:** 


### *Valuable Surgical Discovery.*

PARIS, July 28th, 1859.

A medical discovery of much value, destined to effect a great amelioration in the treatment of ulcers, abscesses, flesh wounds, &c., has lately been made by two former *internes* or house surgeons of the Hospice de la Charite, and by them generously offered to the world without fee or reward. At the last sitting of the Academy of Science M. Velpeau demanded permission to make an important communication, and announced that the two young practitioners in question, Messrs. Crome and Demeaux, had paid him a visit for the purpose of presenting to his notice their discovery and explaining to him its results. Messrs. Crome and Demeaux have found a process for the complete and instantaneous disinfection of animal matter. The action of the disinfecting agent arrests the progress of decomposition, and effectually prevents the generation of insects. The substance, prepared for use, costs here about one franc for a hundred pounds, and the expense in America would probably be still less. The following is the formula, as given by the inventors themselves :

Plaster of commerce, reduced to a fine powder, 100 parts ; coal tar, one to three parts. The mixture of the two substances is effected with ease by the aid of a mortar, or by any other appropriate mechanical means. The application of this composition to the dressing of sores or wounds requires a particular preparation. A certain quantity of the powder, prepared according to the formula, is diluted with olive oil to the consistency of a paste or ointment. This species of paste or salve is of a dark brown color, has a slightly bituminous odor, and may be kept in a closed jar for an indefinite period. The oil unites the powder without dissolving it, and the composition has the property of absorbing infectious liquids the instant it is applied to the sore which produces them. The application may be mediate or immediate. In the latter case, that is to say, placing the composition directly in contact with the sore, no pain whatever is produced; on the contrary, the salve has a detersive action, cleanses the sore, and favors circulation.

In the course of his remarks, M. Velpeau mentioned the case of a patient at the Charitie, to whom the new process had been applied, with perfect success. This person was afflicted with a frightful abscess in the thigh, from which exuded a purulent matter of a most infectious odor, rendering the operation of the Surgeon both painful and diffiicult. This matter, mixed with a powder held in readiness by the two experimentalists, was disinfected in one minute, touched with impunity by the spectators, and applied beneath their noses, without leaving a trace of unpleasant odor.

As has been seen, the elements of this composition are of the simplest character, and though intelligence of the discovery could not have reached the medical faculty of the United States in advance of this letter, your own surgeons will doubtless receive, by the same mail which carries this, every corroborating particular. My desire is to make known the event throughout our country, and I sincerely hope this paragraph may be widely copied by your exchanges. As M. Velpeau himself observed at the close of his observations before the Academy, too much publicity cannot be given to so valuable a discovery, as well as the disinteredness of its authors. In their own report, Messrs. Crome and Demeaux state that the composition may be applied in the form of a poultice, or on cotton, and laid on the wound. They demonstrate that their mode of dressing possesses the double properties of disinfecting morbid products, and of absorbing their liquids. This last circumstance entirely obviates the necessity of lint—which is one of the most important features of the discovery.—*N. Y. Express.*

